# Supplementary material for: On the Dynamics of the Carbon–Bromine Bond Dissociation in the 1-Bromo-2-Methylnaphthalene Radical Anion
Source: Molecules. 2022 Jul 15;27(14):4539. doi: 10.3390/molecules27144539 (PMC9319363; doi:10.3390/molecules27144539)
Supplement: Supplementary file 1 [file molecules-27-04539-s001.zip › molecules-1742818-supplementary.pdf]

SUPPLEMENTARY MATERIALS

On the Dynamics of the Carbon-Bromine Bond Dissociation in the 1-Bromo-2-Methylnaphthalene Radical Anion

Marco Bonechi [1], Walter Giurlani [1], Massimo Innocenti [1,2,3]*, Dario Pasini [4], Suryakant Mishra [5], Roberto Giovanardi [6] and Claudio Fontanesi [2,6]*

[1] Department of Chemistry, "Ugo Schiff", University of Firenze, via della Lastruccia 3, 50019 Sesto Fiorentino, Italy

[2] National Interuniversity Consortium of Materials Science and Technology (INSTM), Via G. Giusti 9, 50121 Firenze, Italy

[3] Center for Colloid and Surface Science (CSGI), Via della Lastruccia 3, 50019 Sesto Fiorentino, Italy

[4] Department of Chemistry, University of Pavia, via Taramelli 10, 27100 Pavia, Italy

[5] Center for Integrated Nanotechnologies, Materials Physics and Applications Division, Los Alamos National Laboratory, Los Alamos, NM 87545, USA

[6] Department of Engineering "Enzo Ferrari" (DIEF), University of Modena, Via Vivarelli 10, 41125 Modena, Italy

** Correspondence: claudio.fontanesi@unimore.it, m.innocenti@unifi.it*

Index

1) Path A PES

2) Path B PES

3) Path C PES

4) Path B DRC

5) Path C DRC

6) Theoretical UV-VIS, IR and CD spectra

1) Path A PES

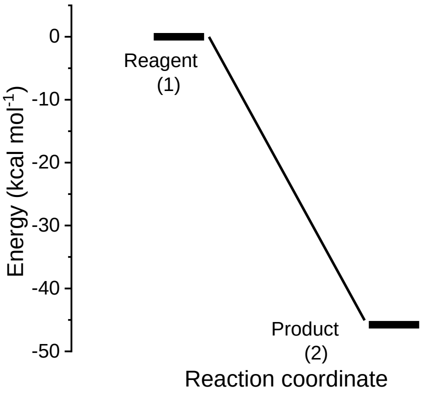

Figure S1. Potential energy surface for reaction path A, at the B3LYP/cc-pVTZ level of theory. Gibbs free energy of reagents (**1**), final products (**2**). The energy levels have been normalized to the energy of state (**1**), which corresponds to a 1882041.187849 kcal mol^{-1} absolute value.

2) Path B PES

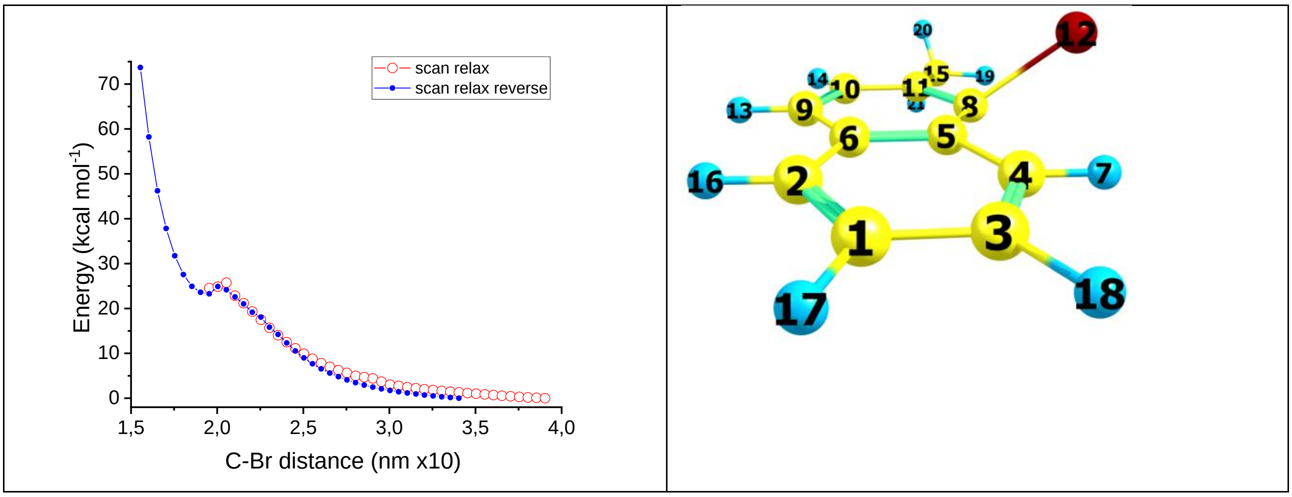

Figure S2. Molecular potential energy vs $C_8 - Br_{12}$ distance for the 1-bromo-2-methylnaphthalene radical anion (2) UB3LYP/cc-pVTZ level of theory, using the Barone and Cossi's polarizable conductor model (CPCM) scan relax.

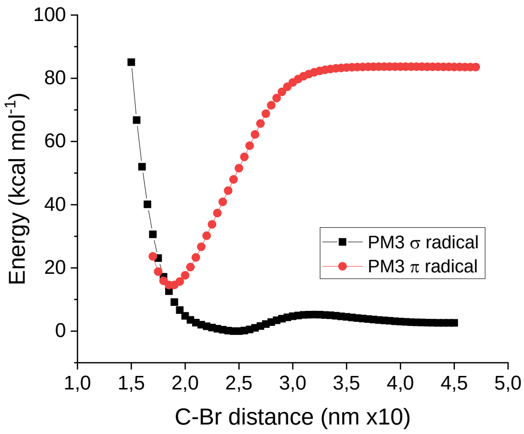

Figure S3. Molecular potential energy vs $C_8 - Br_{12}$ distance, unrelax scan, for the 1-bromo-2-methylnaphthalene radical anion (2). PM3 level of theory, using the Barone and Cossi's polarizable conductor model (CPCM).

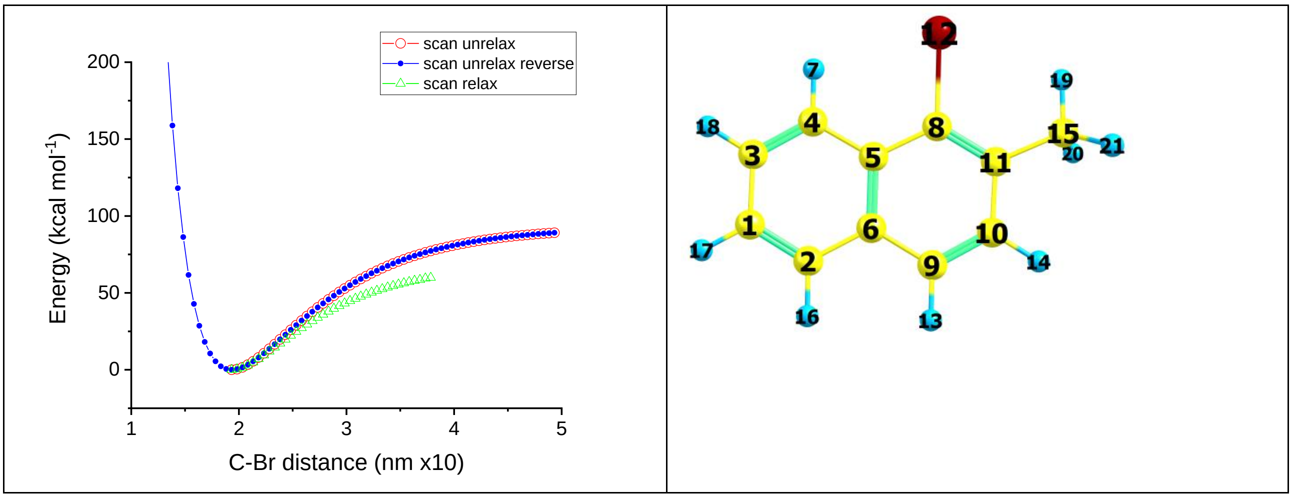

Figure S4. Molecular potential energy vs $C_8 - Br_{12}$ distance for the 1-bromo-2-methylnaphthalene neutral parent species (1), UB3LYP/cc-pVTZ level of theory, using the Barone and Cossi's polarizable conductor model (CPCM)

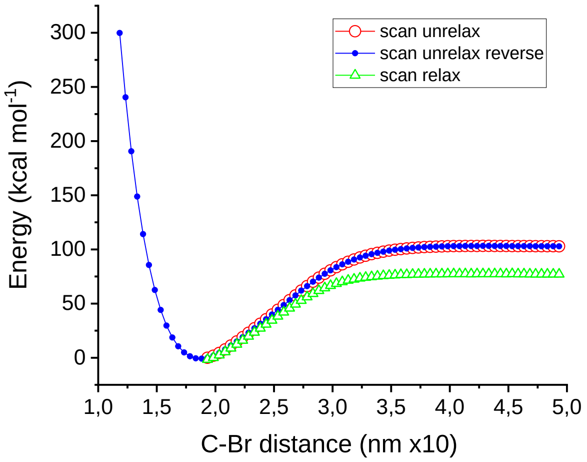

Figure S5. Molecular potential energy vs $C_8 - Br_{12}$ distance for the 1-bromo-2-methylnaphthalene neutral parent species (1) PM3 level of theory, using the Barone and Cossi's polarizable conductor model (CPCM).

3) Path C PES

a)	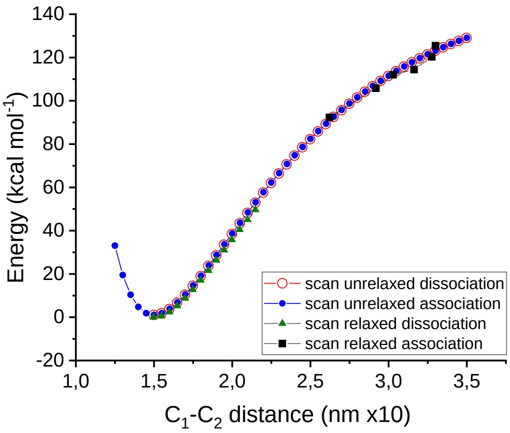
b)	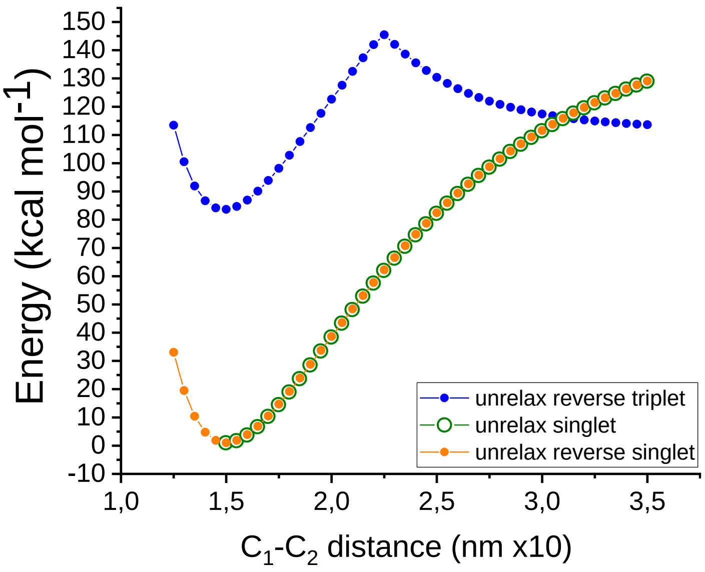

c)

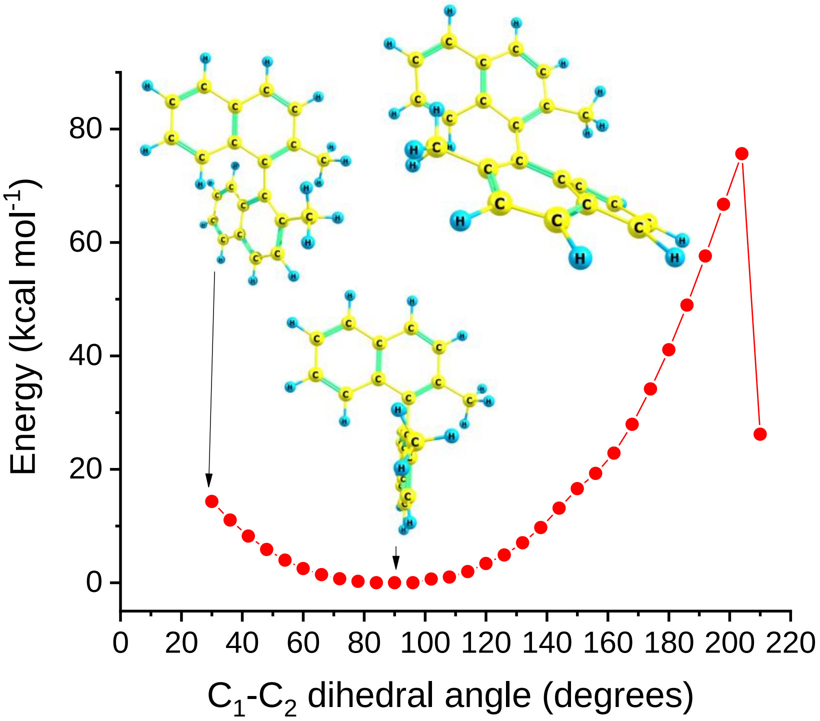

Figure S6. a) Molecular potential energy vs $C_1 - C_2$ distance for the neutral closed shell dimer 1,1'-binaphthalene, 2,2'-dimethyl (4), unrelaxed potential energy surface for the C-C association (UB3LYP/cc-pVTZ level of theory) vs relaxed potential energy surface b) Molecular potential energy vs $C_1 - C_2$ distance for the dimer 1,1'-binaphthyl (4) in the case of spin multiplicity equal to three (triplet state). UB3LYP/cc-pVTZ level of theory, using the Barone and Cossi's polarizable conductor model (CPCM). Overlay of triplet and singlet state curves. c) Molecular potential energy vs $C_1 - C_2$ dihedral angle for the dimer.

4) Path B DRC

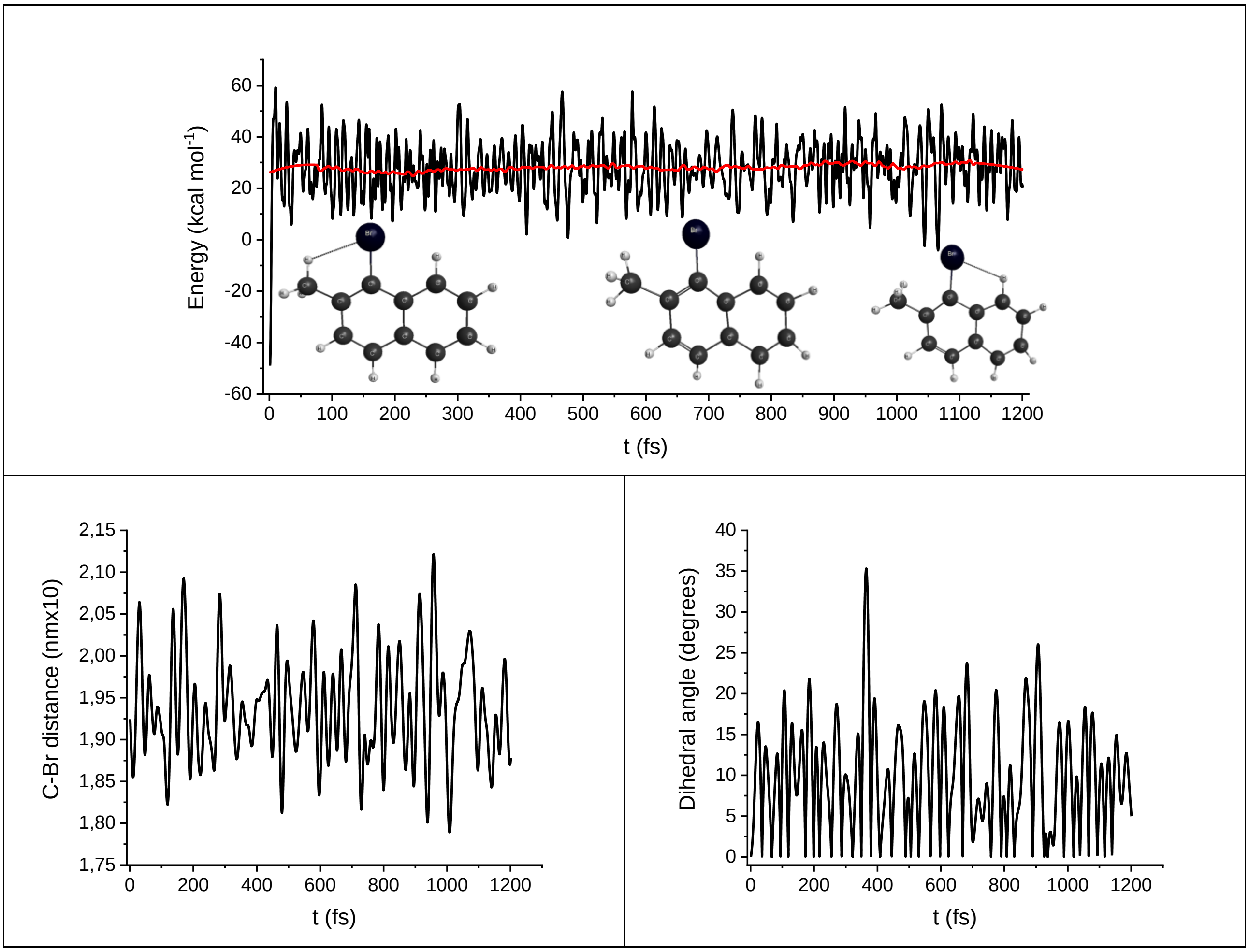

Figure S7. Path B DRC results, B3LYP/6-31G(d), DRC trajectory for the 1-bromo-2-methylnaphthalene neutral parent species (1). a) molecular electronic potential energy vs time b) Carbon bromine distance as a function of time c) dihedral angle between the dissociating $C - Br$ bond as a function of time.

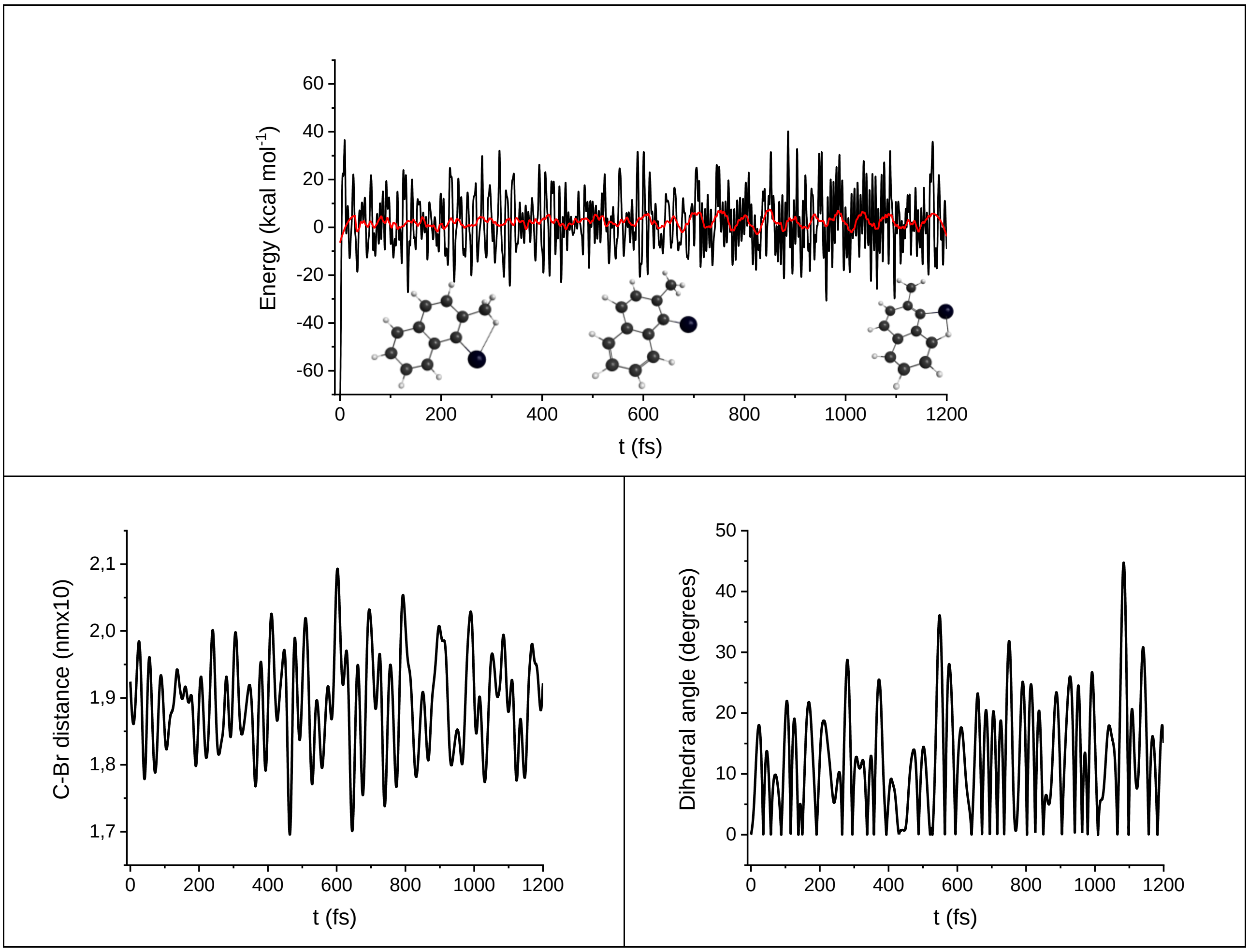

Figure S8. Path B DRC results, PM3 level of theory, DRC trajectory for the 1-bromo-2-methylnaphthalene neutral parent species (1). a) molecular electronic potential energy vs time b) Carbon bromine distance as a function of time c) dihedral angle between the dissociating $C - Br$ bond as a function of time.

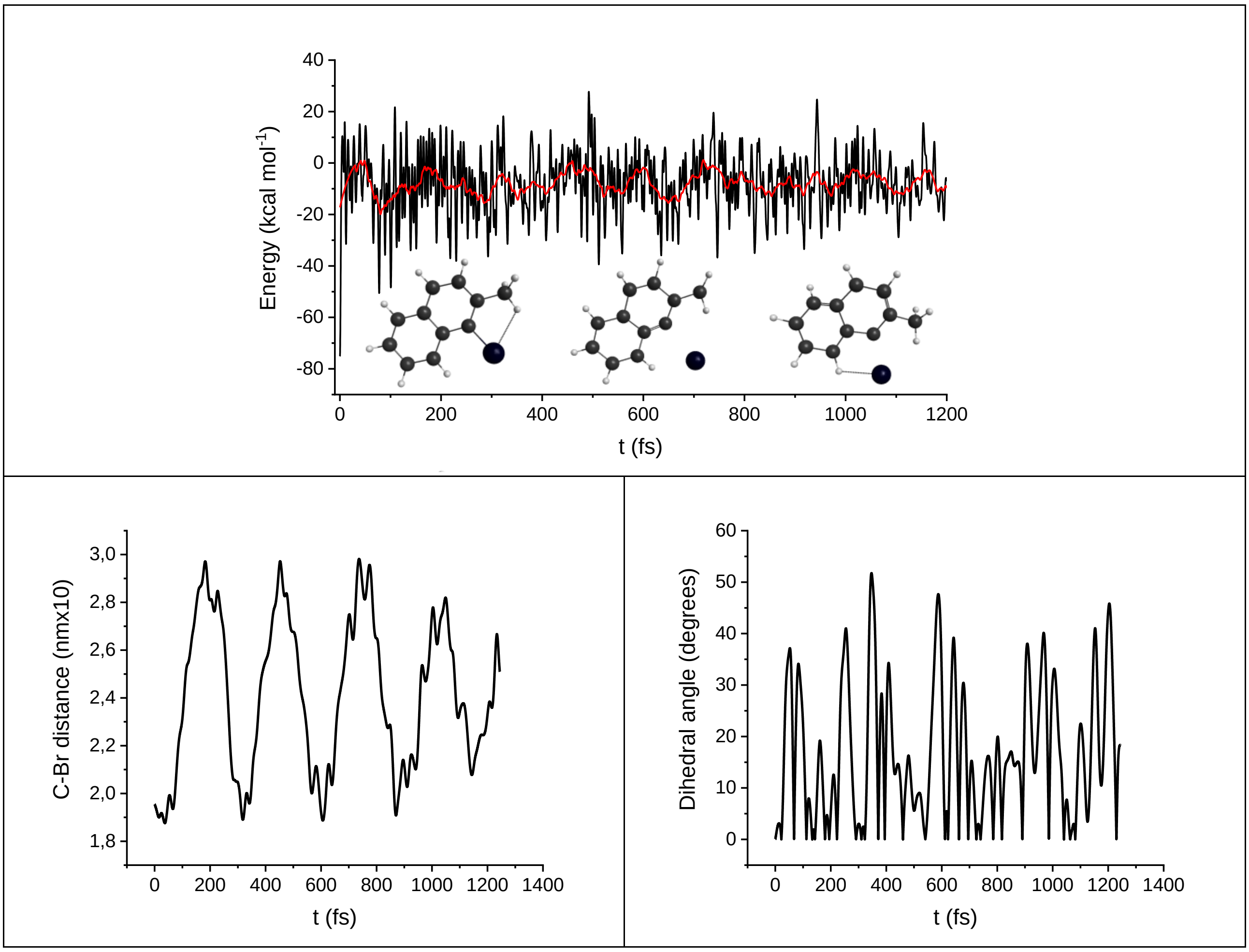

Figure S9. Path B DRC results, PM3 level of theory 1-bromo-2-methylnaphthalene DRC trajectory for the doublet radical anion. a) molecular electronic potential energy vs time b) Carbon bromine distance as a function of time c) dihedral angle between the dissociating $C - Br$ bond as a function of time.

5) Path C DRC

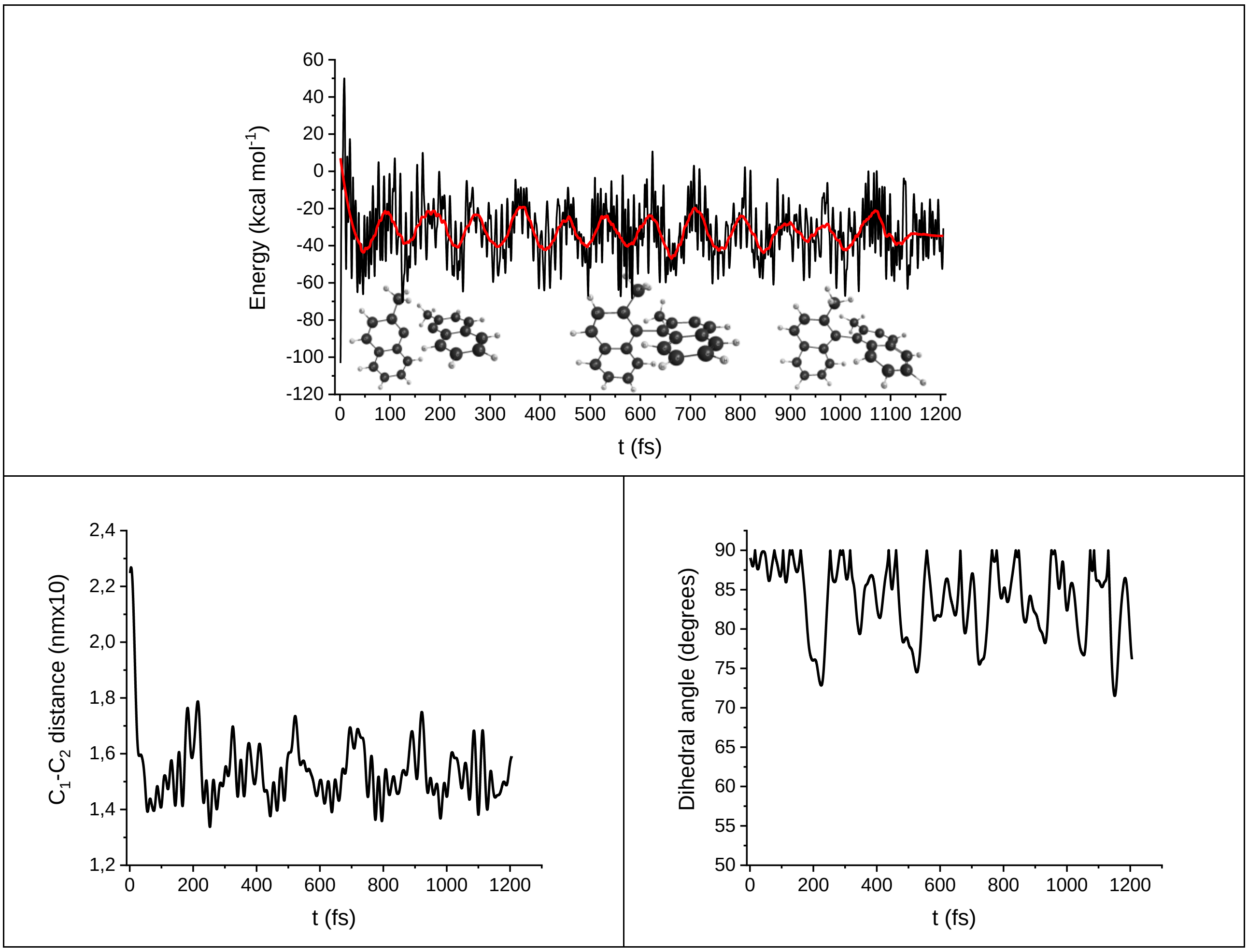

Figure S10. Path C DRC results, B3LYP/6-31G(d) DRC trajectory for the coupling of two 1-Methylnaphthalene (3) radical forming 1,1'-Binaphthalene, 2,2'-dimethyl (4) from a distances of 2.5 Amstrong. a) molecular electronic potential energy vs time, b) $C-C$ bond between the two aromatic rings distance as a function of time c) dihedral angle between the dissociating $C-Br$ bond as a function of time.

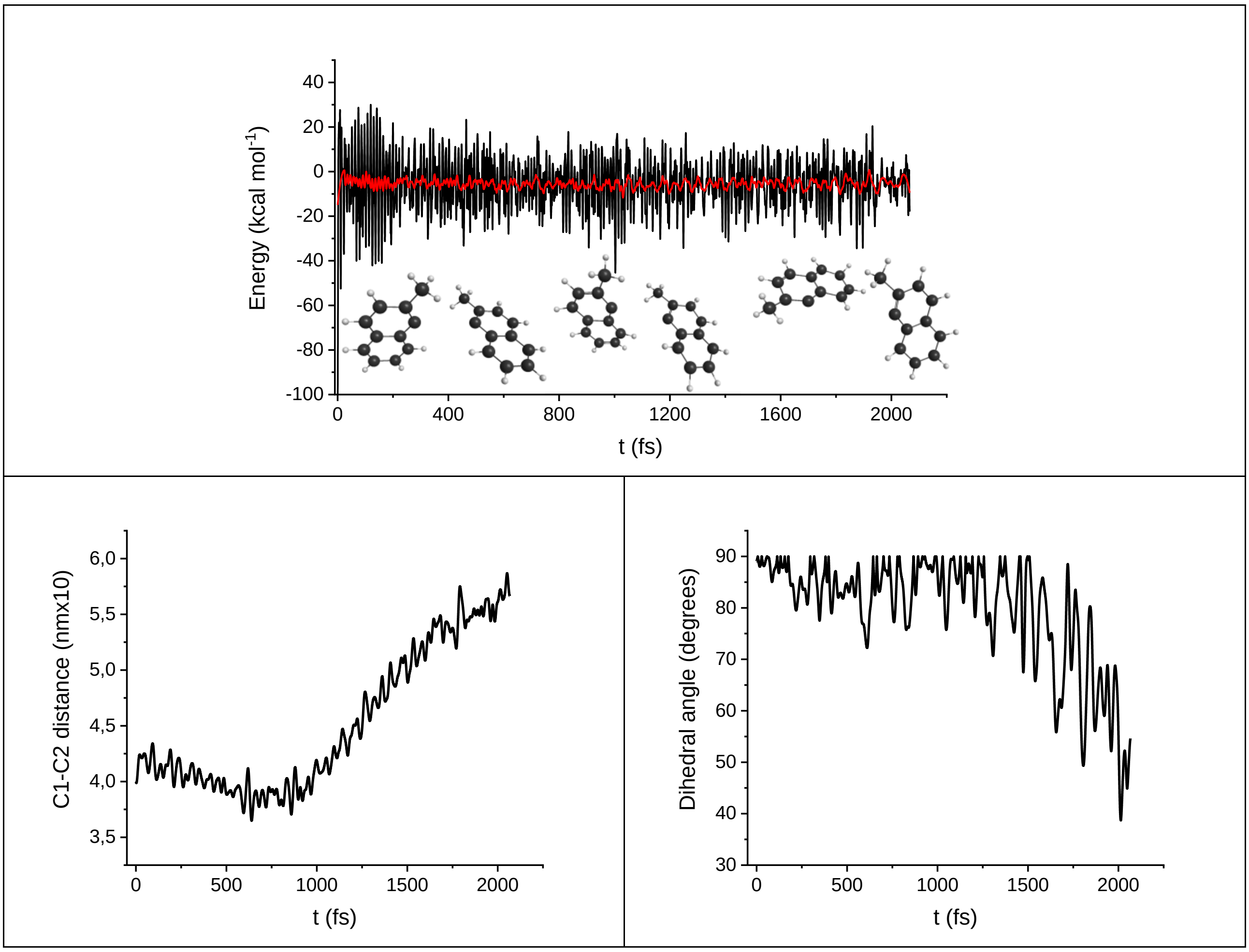

Figure S11. Path C DRC results, B3LYP/6-31G(d) DRC trajectory for the coupling of two 1-Methylnaphthalene (3) radical forming 1,1'-binaphthalene, 2,2'-dimethyl (4). $(3)^{\alpha\cdot} + (3)^{\alpha\cdot} \rightarrow (4)$. a) molecular electronic potential energy vs time, b) $C-C$ bond between the two aromatic rings distance as a function of time c) dihedral angle between the dissociating $C-Br$ bond as a function of time.

6) Theoretical UV-VIS, IR and CD spectra

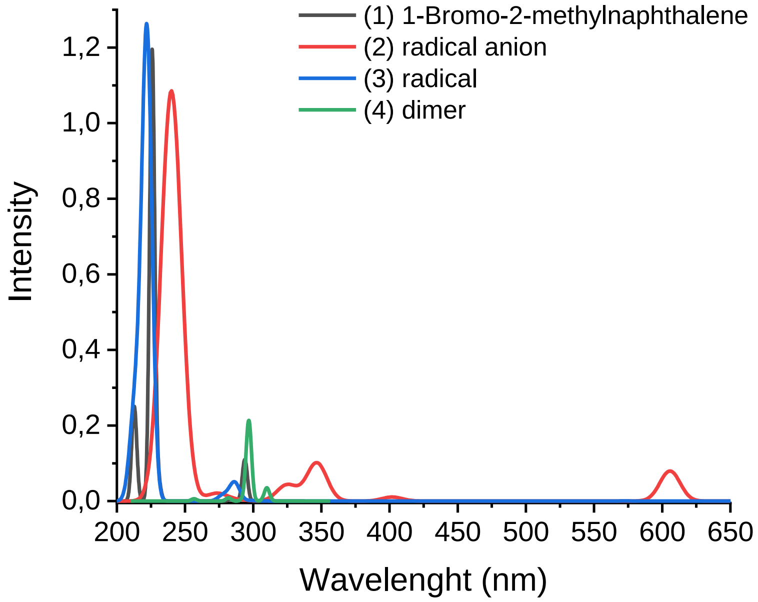

Figure S12. UV-VIS spectra calculated at the cc-pVTZ level of theory, in the wavelength range between 200 and 650 nm of all the species under study (1)-(4).

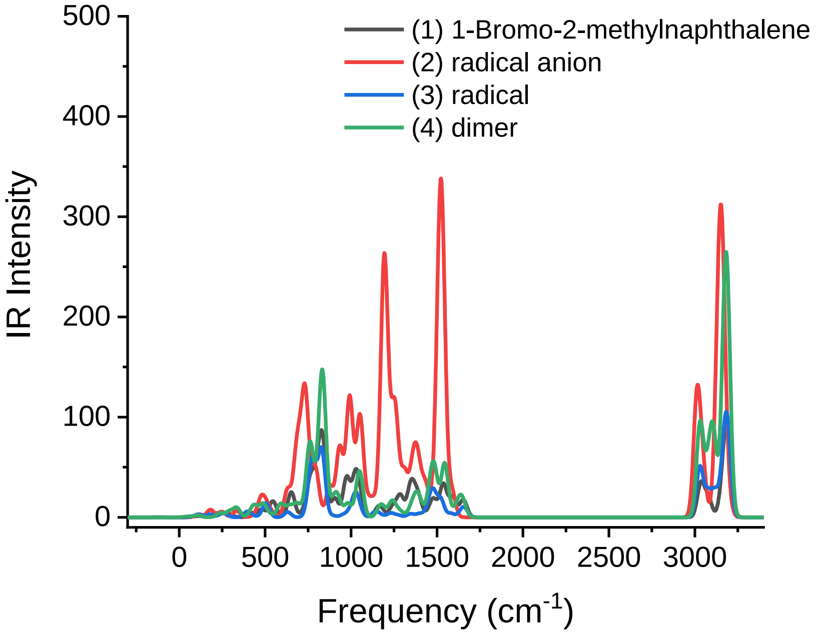

Figure S13. IR spectra calculated at the cc-pVTZ level of theory, in the wavelength range between 0 and 3500 cm^{-1} of all the species under study (1)-(4).

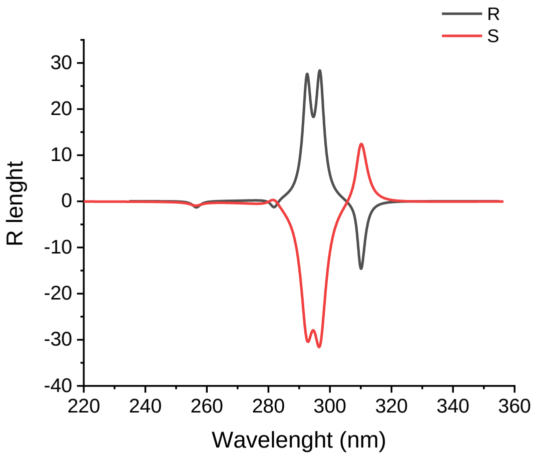

Figure S14. CD spectra calculated at the cc-pVTZ level of theory, in the wavelength range between 220 and 360 nm of (4)-R and (4)-S structures.

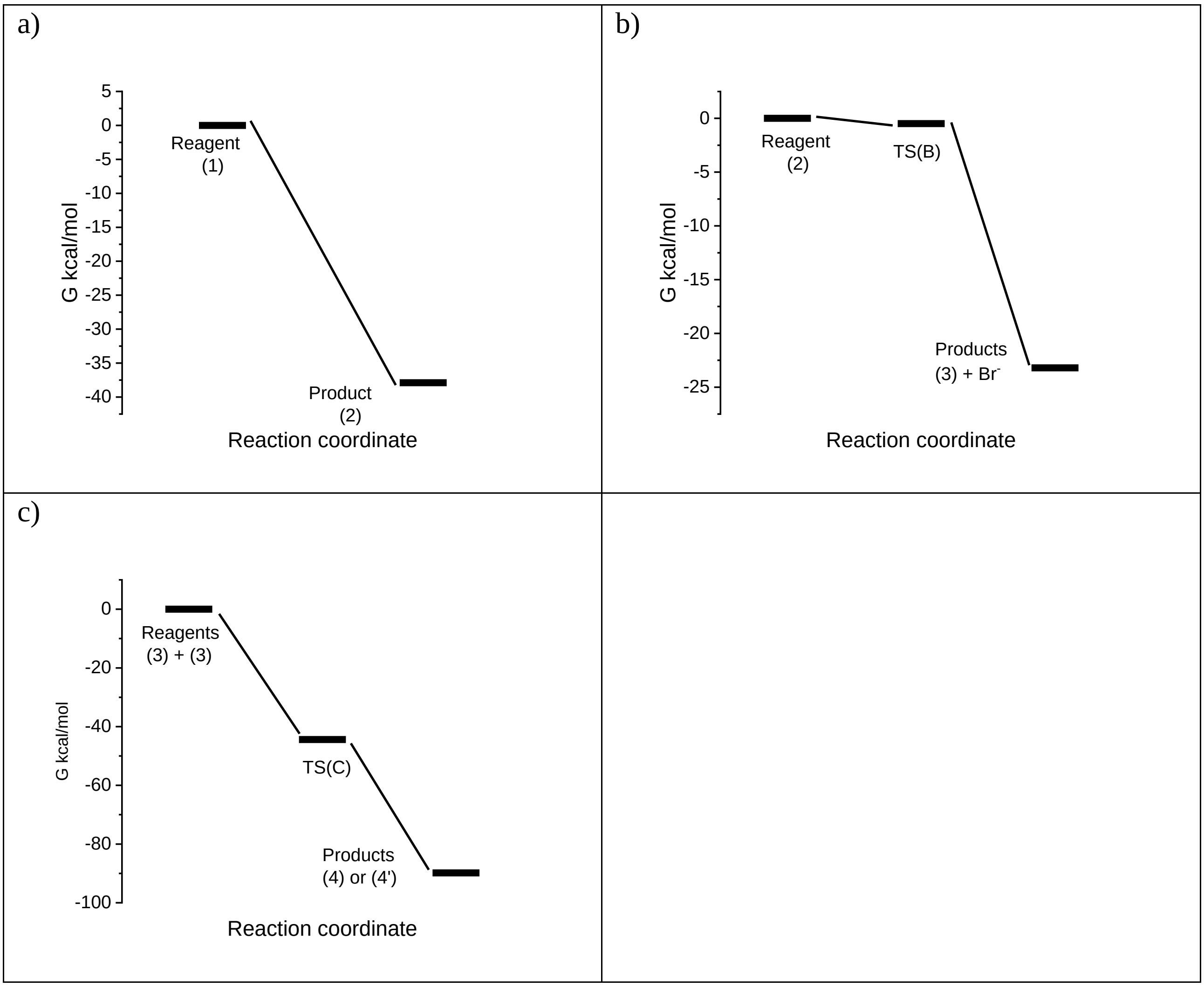

Figure S15. a) Potential energy surface for reaction path A, at the B3LYP/6-31G(d) level of theory. Gibbs free energy of reagents (**1**), final products (**2**). b) Potential energy surface for reaction path B, at the UB3LYP/6-31G(d) level of theory. Gibbs free energy of reagents (2), final products ($3 + Br^{-}$) and transition state ($TS(B)$) c) Potential energy surface for reaction path C, at the UB3LYP/6-31G(d) level of theory. Gibbs free energy of reagents (3), final products (4) and transition state ($TS(C)$).
